# Effects of Microplastics on Gene Expression, Muscular Performance, and Immunological Responses in Nile Tilapia (*Oreochromis niloticus*): Seasonal and Habitat Variations

**DOI:** 10.1007/s10126-025-10481-6

**Published:** 2025-06-27

**Authors:** Noura M. Nabawy, Seham A. Ibrahim, Nassr Allah Abd El-Hameid, Omar I. Ghonemy, Walaa M. Shaalan

**Affiliations:** Department of Zoology, Faculty of Science, Benha University, Benha, 13518 Egypt

**Keywords:** Nile tilapia (*Oreochromis niloticus)*, MPs, Muscle-related genes, Immune-related genes, FTIR, Histology, Polarizing microscope, Seasons

## Abstract

Microplastics (MPs; less than 5 mm in size) are becoming increasingly prevalent in both terrestrial and aquatic ecosystems. As these particles enter the food chain, they have the potential to pose significant risks to human health. However, their effects on vital fish tissues, such as skeletal muscle, are not yet fully understood. In this study, we examined Nile tilapia (*Oreochromis niloticus*) from two distinct sites on the Nile River in Egypt: the Nile branch (Damietta branch) and Riah El-Towfiqi. Using Fourier Transform Infrared Spectroscopy (FTIR) and histological study, we confirmed the presence of MPs in both gastrointestinal and muscle tissues. We focused on understanding how MPs might affect fish muscle by investigating the expression of genes involved in muscle atrophy and hypertrophy using Real Time-PCR and histological alterations in muscle tissues of tilapia collected from the two studied sites in the four seasons. Our results revealed histological alterations in muscle tissues collected from the two sites studied in the four seasons. The expression levels of atrophy-related genes, *Atrogin-1* (*Fbxo32*), *Capn-1*, and the apoptosis marker *Caspase3a* (*Casp3a*), showed increased expression, especially during the summer at both sites. On the other hand, the hypertrophy-related gene *Igf-1* exhibited a significant decrease while, muscle stem cell genes (*Pax3*, *Pax7*) and muscle differentiation gene markers (*Myf5*, *Mrf6*) displayed seasonal upregulation, with heightened activity during winter and summer, depending on the location. Additionally, immune-related genes (*Ccr9*, *Irak4*, *Igl-1*, *Tlr1*) demonstrated notable seasonal changes, with a peak during summer at the Nile branch. These findings demonstrate that MPs can disrupt muscle integrity and immune function in fish, with implications for ecosystem health and potential risks to human food security.

## Introduction

Fish provide a primary source of animal protein, with approximately half of the global population relying on it for sustenance (Viana et al. 2023). Nile tilapia (*Oreochromis niloticus*) is a primary food source in Africa, America, and Asia, attributed to its smooth flesh, exceptional flavor, rapid growth, adaptability to diverse environmental conditions, ease of breeding, and disease resistance (El-Sherif and El-Feky 2009). The rising population has resulted in significant increases in urbanization, industry, and agricultural land use, leading to extensive pollutant discharges into various water resources, which adversely affect the aquatic environment (Elsayed et al. 2003). A further environmental pollutant identified is microplastics, MPs (< 5 mm in diameter), (Carpenter et al. 1972; Colton Jr 1974; Gregory 1978). Earlier research indicated that 21% of utilized plastics are either repurposed or incinerated, while the remainder degrades into small fragments due to the effects of sunshine, wind, and wave action, resulting in MPs (Bajt 2021; Scotti et al. 2023). Recently, Khan et al., (2020) revealed the presence of MPs as pollutants in the GIT of various fish species in the River Nile, Egypt. Furthermore, numerous studies have identified the translocation of MPs from the gastrointestinal tract (GIT) to various tissues, including the liver of *Mugil cephalus* (size ~ 200–600 μm), *Engraulis encrasicolus* (size ~ 39–90 μm), and *Sparus aurata* (size ~ 214–288 μm) (Avio et al. 2015; Collard et al. 2017; Jovanović et al. 2018), as well as in the muscles of *Platycephalus indicus*, *Saurida tumbil*, *Sillago sihama*, and *Cynoglossus abbreviatus* (Abbasi et al. 2018). Moreover*,* the presence of MPs in different tissues of the organism was observed using polarized and light microscopes (Pittura et al. 2018; Hamed et al. 2021). Pittura et al., (2018) used the polarized-light microscope to visualize and determine the position of low-density polyethylene (LDPE) microplastics in stained H&E histological sections of Mediterranean mussels, *Mytilus galloprovincialis,* where MPs were found in hemolymph, gills, gut lumen, epithelium and digestive tubules.

*Atrogin-1* (*Fbxo32*) is a member of the forkhead box subfamily proteins, identified as a pivotal factor in skeletal muscle atrophy (Bodine et al. 2001; Gomes et al. 2001; Powell et al. 2012; Shaalan et al. 2023). It is an E3 ligase within the ubiquitin–proteasome system (UPS) in skeletal muscles that facilitates the polyubiquitination of proteins, thereby tagging them for degradation by the 26S proteasome (Yoshida and Delafontaine 2020). Calpain enzymes are non-lysosomal, calcium-activated cysteine proteases found in muscle tissues, indicating that their activation relies on Ca^+2^ binding to conserved amino acids (Ono and Sorimachi 2012; Shaalan et al. 2023). *Calpain1 (µ-calpain)*, *Capn-1,* a prevalent enzyme within the calpain family (Smith et al. 2008), is observed to be elevated during muscle degeneration (Smith et al. 2008). Cysteine-dependent aspartate proteases, referred to as caspases, are pivotal in the apoptosis process, facilitating normal development and maintaining homeostasis in multicellular organisms by eliminating atypical and redundant cell types without affecting adjacent normal cells. This mechanism resulted in significant apoptosis in various organs of adult transgenic zebrafish with overexpression, including skeletal muscle fibers (Takle and Andersen 2007). Insulin-like growth factor-1 (*Igf-1*) is an essential growth factor that regulates anabolic and catabolic pathways in skeletal muscle (Yoshida and Delafontaine 2020). It enhances skeletal muscle protein synthesis through the phosphoinositide-3-kinase (PI3K)/protein kinase b (AKT/mammalian target of rapamycin (mTOR)/Glycogen Synthase Kinase 3 Beta (GSK3β) pathways, while also inhibiting the forkhead box subfamily proteins, leading to a reduction in E3 ligase transcription that regulates the ubiquitin–proteasome system via the PI3K/AKT route (Yoshida and Delafontaine 2020).

Pax genes, specifically *Pax3* and *Pax7*, play a crucial role in embryonic development. Both genes originated from the duplication of the unique ancestral *Pax3/7* gene, which explains the notable resemblance in their activities and expressions (Relaix et al. 2004). The *Pax3* gene plays a crucial function in skeletal muscle development (Buckingham and Relaix 2015). *Pax7* is a paralogue of *Pax3;* mutant *Pax7* mice exhibited an absence of satellite cells necessary for the growth and regeneration of skeletal muscles, underscoring the significance of *Pax7* expression in the differentiation of adult muscle progenitor cells (Seale et al. 2000). Guo et al., (2009) and Wu et al., (2021) documented that *Myf5* is a member of the myogenic regulatory factors (MRFs) family, which also comprises *MyoD*, myogenin, and myogenic factor 6 (*Myf6, Mrf4*). These regulatory factors dictate the destiny of skeletal muscle precursor cells, as they possess a basic helix-loop-helix (bHLH) domain that enables their interaction with E-proteins (E-box), resulting in the formation of protein dimers (Sun and Baltimore 1991). *Myf5* is the inaugural expressed gene from the MRF family, found in early somites and myotomal muscle (Braun et al. 1992). *Mrf6*, also known as myogenic regulatory factor 4 (*Mrf4*) (Zhou et al. 2018), that contributes to muscle differentiation in fish by promoting myotube maturation (Johnston 1999; Wu et al. 2021).

MPs have been identified as contributors to oxidative stress in fish, resulting in the generation of reactive oxygen species that might trigger immunological responses (Schieber and Chandel 2014; Subaramaniyam et al. 2023). Chemokine receptors (CRs) are pivotal elements in regulating cellular responses throughout several biological processes (Su et al. 2019). They are designated based on the class of chemokines to which they bind; for instance, CCRs bind to CC-chemokines (Grimholt et al. 2015). In teleost fish, the binding of CCR9 to its ligand CCL25 has been shown to initiate an inflammatory response by attracting additional CCR9-expressing T cells to the inflammation site, driven by elevated levels of CCL25 (Galindo-Villegas et al. 2013). *Tlr1* is a constituent of the toll-like receptor family (TLRs). They are the most often documented receptors in the innate immune system that identify many types of infections in both vertebrates and invertebrates (Palti 2011). The TLR signaling pathways stimulate the synthesis of proinflammatory cytokines, including interleukin (IL), tumor necrosis factor (TNF), and type I interferon (IFN), which collectively attract various immune cells to the site of infection (Iwasaki and Medzhitov 2004; Kawai and Akira 2010).

Interleukin-1 receptor-associated kinases (IRAKs) are essential signaling molecules in immune inflammatory responses facilitated by TLR/IL-1R (Han et al. 2020). They identified two IRAK members in Nile tilapia (*Oreochromis niloticus*), namely *Onirak1* and *Onirak4* (Han et al. 2020). *Irak4* plays a crucial function in the innate immune system, since its deficiency results in bacterial infections (Picard et al. 1977; Suzuki et al. 2002). The *Igl-1* gene is involved in the formation of the pre-B cell receptor (pre-BCR), which is essential on the cell membrane for signal transduction and immunoglobulin gene rearrangements, facilitating the survival and production of pre-B cells (Gemayel et al. 2016; Minegishi et al. 1998; Spanopoulou et al. 1994; Young et al. 1994). A mutation in the *Igl-1* gene may result in a deficit of B lymphocytes (Conley et al. 2009; Gemayel et al. 2016; Minegishi et al. 1998).

This study aimed to examine the impact of MPs on the expression of muscle-related and immune-related genes in Nile tilapia (*Oreochromis niloticus*) collected seasonally from two tributaries of the Nile River: the Damietta branch and the Riah El-Towfiqi. The selected genes include key markers of muscle atrophy (*Atrogin-1* (*Fbxo32*), *Capn-1*), and apoptosis marker gene *Caspase3a* (*Casp3a*). Also, muscle hypertrophy gene (*Igf-1*), and muscle stem cell genes (*Pax3*, *Pax7*). Additionally, muscle differentiation gene markers (*Myf5*, *Mrf6*). Moreover, immune related genes (*Ccr9*, *Irak4*, *Igl-1*, *Tlr1*) which are commonly associated with physiological stress, inflammation, and tissue remodeling in fish. Although not all of these genes have been previously studied in the context of microplastic exposure, related research has shown that MPs can cause oxidative stress, growth inhibition, apoptosis, and immune disruption in aquatic organisms (Romano et al. 2020; Tang 2025). Thus, the present study investigates whether seasonal MP exposure correlates with molecular changes in these gene pathways, potentially affecting fish health and quality.

## Materials and Methods

### Study Area and Fish

**Fig. 1:**
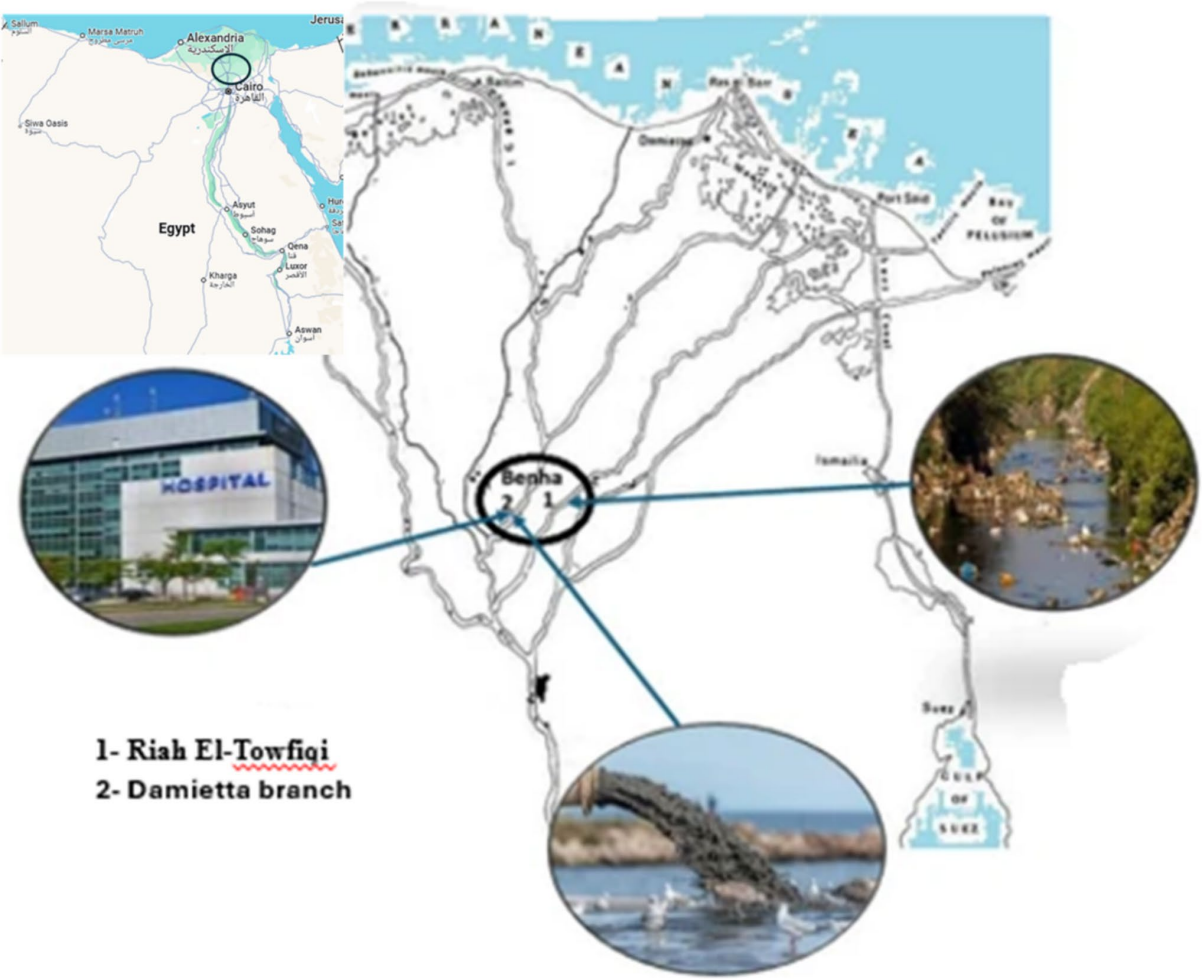
The map shows the two sites of sample collection from the Damietta branch and Rah El-Towfiqi at Benha, Al-Qalyubiya, Egypt. Modified from (Shaalan 2024)

This study received approval from the Zoology Department's animal care and use committee, under protocol number (ZD/FSc/BU-IACUC/2024-25). The study commenced in August 2022 and persisted for an entire year. Fifteen specimens of Nile tilapia (*Oreochromis niloticus*) were seasonally obtained by fishermen from each of the two previous mentioned locations in Benha, Al-Qalyubiya, Egypt, Fig. 1. The fish were gathered and brought alive in containers to the laboratory, where they underwent anesthetization with MS222 (Syndel USA, Ferndale, WA) prior to dissection. The gastrointestinal tract, GIT, of each fish was excised on the same day of arrival at the laboratory for digestion. The muscle samples were preserved at −80°C for molecular analysis. Prior to the dissection, all fish were measured and weighed. Throughout the four seasons, the Nile tilapia samples exhibited an average weight of 280.9 ± 75.05 gm and an average length of 25.66 ± 2.07 cm.

### Tissue Digestion and MPs Extraction

The fish were dissected, and the gastrointestinal tracts were processed for MPs extraction as previously described (Biginagwa et al. 2016). The whole GIT of each fish was excised following a longitudinal incision in the abdomen. The dissected tissues were subsequently immersed in 250 mL beakers containing a 10% sodium hydroxide (NaOH) solution, added at a 5:1 (w/v) ratio. The NaOH digestion (75 °C for 1 h) was conducted to extract MPs from the organic tissue. This approach demonstrates efficiency exceeding 90% with NaOH (Cole et al. 2014; Zhao et al. 2014) and does not adversely affect the existing MPs, such as causing discoloration or digestion. After digestion, each digested sample was subjected to filtration utilizing double-ring filter paper (Grade Medium 102, 15 cm diameter, China). The filter sheets were placed in a dry, sterile hood for drying. Upon thorough drying, each filter paper was examined using a light dissecting microscope (40X) (Optica, Ponteranica, Italy). MPs were visually identified, enumerated, and categorized by color, such as vibrant blue or red, and by shape, including irregular, non-segmental filaments, fragments, or intact foam that remains unbroken when manipulated with forceps, flexible, and not fragile (Hidalgo-Ruz et al. 2012; Horton et al. 2018; Mohamed Nor and Obbard 2014; Nel et al. 2018; Tanaka and Takada 2016).

After that, MPs were photographed using an ISCapture 4.1.2 camera (TUCSEN Co., China) connected to a light dissecting microscope. Subsequently, the extracted MPs were examined using Fourier transform infrared spectroscopy (FTIR) (Bruker Co. Ltd., UK) to validate the optical recognition via their chemical structure at a resolution of 2 cm − 1 within the range of 4000 to 400 cm − 1 (Khan et al. 2020).

The frequency of occurrence (FO%) of MPs denotes the percentage of samples containing MPs, computed using the formula FO% = (n_i_/n) × 100, where n_i_ represents the number of samples, (15 samples/season), having MPs and n signifies the total number of investigated samples (Pegado et al. 2018).

### Hisological Study

Muscle samples were washed and fixed in 10% neutral buffered formalin, then dehydrated, cleared, embedded in paraffin wax, cut (5 μm thickness) with microtome and mounted on slides. These slides were stained with Harris’s hematoxylin and eosin, (Lillie and Fulmer 1976), and examined using an Olympus microscope (BX51, Olympus Optical Co. LTP, Japan).

### Molecular Study

Primers for *Atrogin-1, Igf-1, Pax3, Pax7, Myf5, Ccr9, Irak4, Igl-1, Capn-1, Tlr1, Mrf6, Casp3a, and β-actin*, together with the complementary DNA (cDNA) sequences of each gene, were developed utilizing primer3 software (Kõressaar et al. 2018). For normalization of gene expression, β-actin was used as the internal reference gene, (Yang et al. 2013), due to its common application in qPCR studies involving fish, including *Oreochromis niloticus*, and previous validation under various environmental stressors. To confirm its suitability in the context of microplastic exposure, Ct values of β-actin were analyzed across all experimental groups. The results showed no significant variation (*p* > 0.05) in β-actin expression between seasons or habitats, supporting its use as a stable reference gene under our study conditions. The cDNA NCBI accession numbers are listed in Table 1. Total RNA was extracted from muscle and spleen tissues with TRIZOL reagent in accordance with the manufacturer's guidelines. One ml of TRIZOL reagent was incorporated into 50–100 mg of each tissue specimen, which was subsequently homogenized. One microgram of RNA was reverse transcribed into complementary DNA with a cDNA synthesis kit (Thermo Scientific, Hudson, NH, USA). Quantitative Real-Time PCR, (qPCR), was performed in duplicates for each gene of interest using SYBR Green dye (Thermo Scientific, Hudson, NH, USA), and gene expression levels were quantified using Rotor-Gene Q Series Software 2.0.3 (Corbett Life Science, QIAGEN, Hilden, Germany). Each sample was first denatured at 95˚C for 10 min, followed by 45 cycles consisting of denaturation at 95˚C for 10 s, annealing at 60–62°C for 15 s, and extension at 72˚C for 30 s (Shaalan 2024).

**Table 1:** Primers designed for real time-PCR

Genes	Forward primers	Reverse primers	Accession number
*Atrogin-1*	ATGCCTTTTCTCGGACAGGA	GTCGTCGGCTGTTGTCTTTT	XP_003443717.1
*Igf-1*	CACCCTCTCACTACTGCTGT	CACAGTACATCTCAAGGCGC	XM_019346352.2
*Pax3*	AAAGGCGACGAAGAAGAGGA	TGTGTTCTTTCAAAGGCCCG	XM_005461712.3
*Pax7,X1*	GAGATCCGGGATAAGCTGCT	TGAAGCATCATCTGTCCGGT	XM_005459001.4
*Myf5*	TGTGCTTCCTCTCCAGACAG	ACGCCTCAAAGCCTCAAAAG	XM_005456634.3
*Ccr9*	GTGTCATCCTTGCTGTGGTG	AGTCCTCACCATCCTTTGCA	NC_031982.2
*Irak-4*	GTGTACGCTTTCATGGCCAA	TGCTCTCGTCAGACCAAAGT	XM_003443911.5
*Igl-1*	TCCAGTCCAACACAGCTTCT	CTCTGAAGTCTGTGAGCCCA	XM_025906291.1
*Capn-1*	GATCCGAAAGTGGCTGGTCA	TTCCACAGCAAGTCGCATCT	XM_003447550.3
*Tlr1*	GTCTTACAGCCAGCACGATG	ATGACACCACTCGCTCTTGA	MZ210061.1
*Mrf6*	AAGACCGTAGCCAATCCCAA	GACTCACTGGCTCCTTCTGT	NM_001282891.1
*Casp3a*	CACACAGCTTCAGATACCGC	AGGCTGAGTTGCTGTGATCT	NM_001282894.1
β-actin	TCTCGGCTGTGGTGGTGAA	GACCCACACAGTGCCCATCT	XM_003455949

### Statical Analysis

MPs sizes were measured using *ImageJ* software and they were calculated as mean ± SD. Frequency of occurrence (FO%) of MPs was calculated. The fold change was determined using the (2^−ΔΔCt^) method with normalization to β-actin rRNA (Yang et al. 2013).The statistical analysis of the data was done using student unpaired *T.test* using Microsoft excel.

## Results

### Identification and Characterization of MPs

**Fig. 2:**
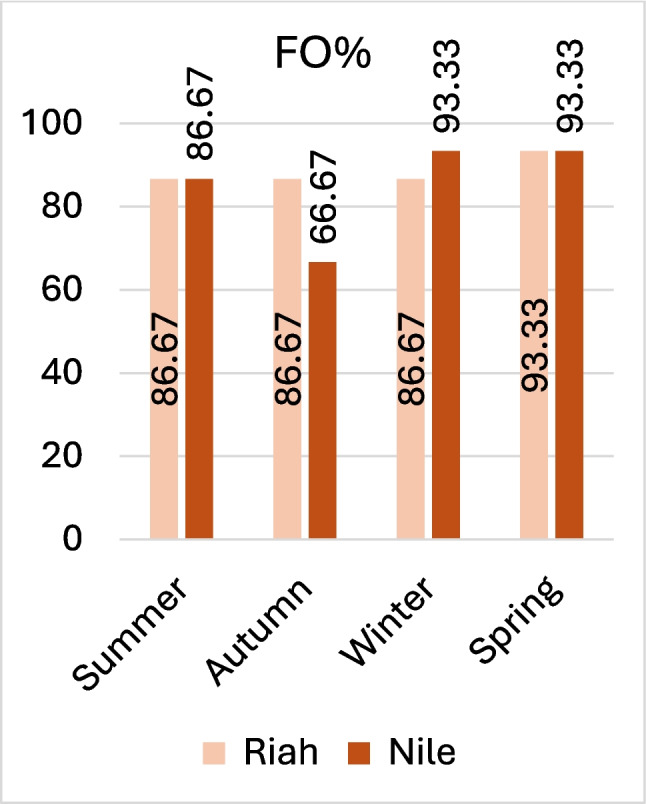
FO% of MPs during the four seasons at the two sites

The current study demonstrated that 86.66%, (104 samples), of the overall 120 samples exhibited MPs contamination in their GITs, and only 16 samples remaining uncontaminated 13.30%. From these 104 samples, 551 MPs were extracted. The maximum FO% of MPs, 93.33% in Nile tilapia fish, was recorded during the winter for Nile branch followed by 86.66% during spring season for both habitats, Fig. 2.

**Fig. 3:**
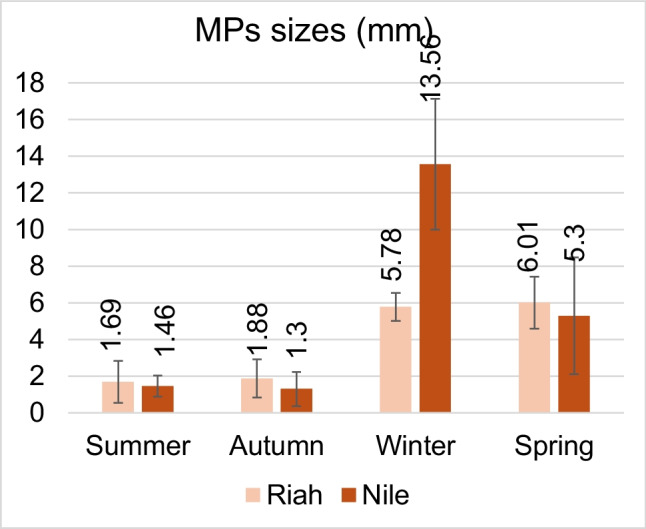
Sizes of MPs during the four seasons at the two tested sites

**Fig. 4:**
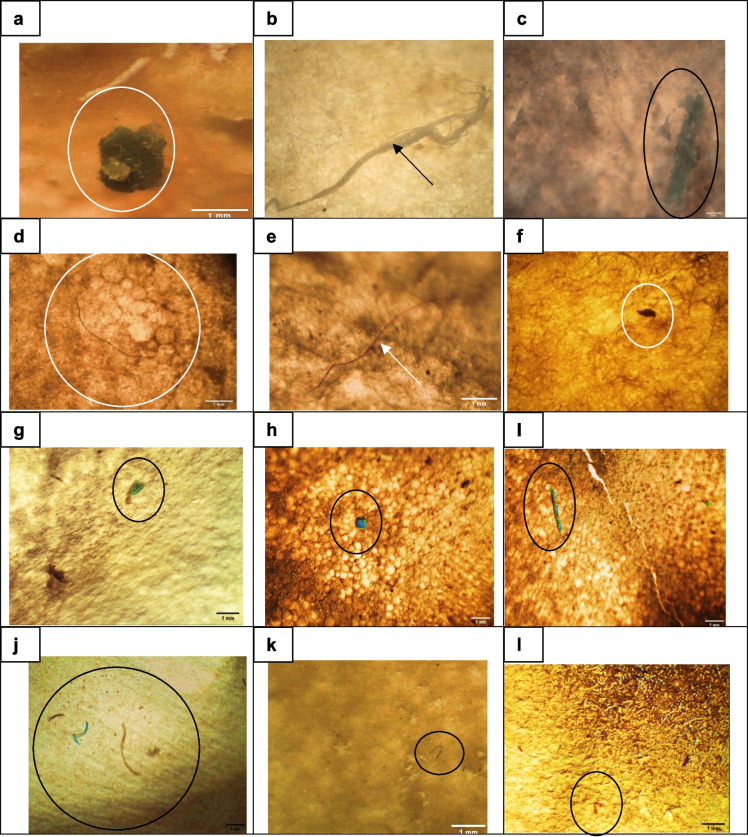
Photomicrograph shows different shapes and colors of MPs (≤ 1 mm) that were found in the GIT of *Oreochromis niloticus*, **a**. dark blue fragment, **b**, white filament **c**. green fragment, **d**. black filament, **e**. red filament, **f**. black fragment, **g**. green pellet, **h**. blue pellet, **i**. green rod, **j**. dark brown, blue and brown filaments, **k**. black filament, **l**. red filament

**Fig. 5:**
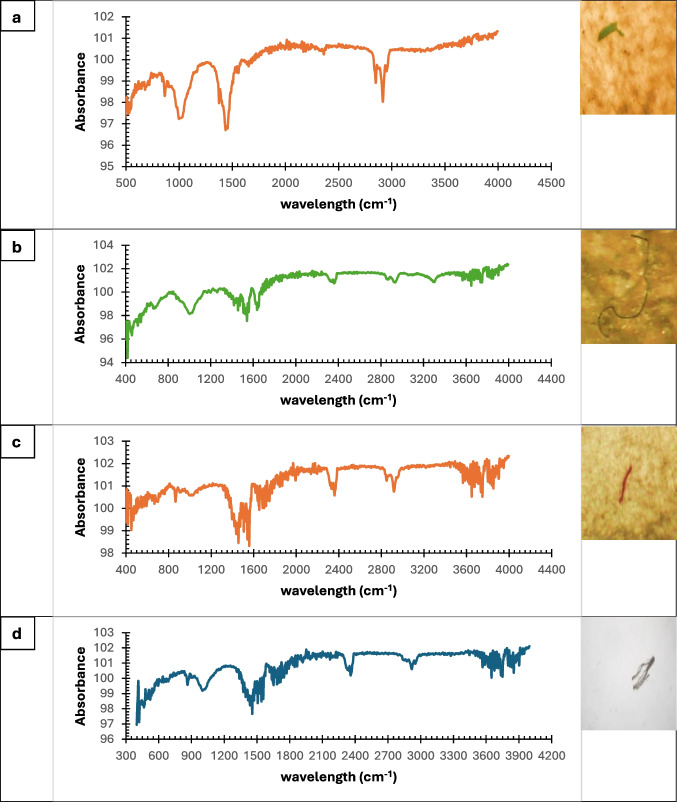
Shows different shapes and colors of MPs and its Fourier-transform infrared (FTIR) spectra of random samples from the four seasons; **a**. black fragment of summer, **b**. black filament of winter, **c**. red filament of autumn and d. white fragment of spring

The present findings revealed that filament persistence in the GIT was 79.85%, fragments accounted for 15.24%, while other forms, including foams, pellets, and irregular shapes, comprised approximately 4.71%. The lengths of extracted MPs from samples of the Riah El-Towfiqi were 1.69, 1.88, 5.78, and 6.01 mm, while those from the Nile branch measured 1.46, 1.30, 13.56, and 5.30 mm during summer, autumn, winter, and spring, respectively, as illustrated in Fig. 3, (data represent mean ± SD). The predominant colors of MPs in filament type were black, followed by blue, red, and white. On the other hand, in fragment type, the order was black, blue, and other colors such as brown, pink, or yellow, as illustrated in Fig. 4, and their FTIR analysis in Fig. 5 and Table 2.

**Table 2:** According to (Rodriguez-Seijo et al. 2017), MPs bands are identified using FTIR-ATR as mentioned in the following table

Wavelength (cm-1)	Band identification
3650–3000	O–H and N–H vibrations
3000–2810	CH2 symmetrical stretch (peak at 2851 cm-1) asymmetrical stretching of methylene
	groups (peak at 2918 cm-1)
1800–1460	Proteins with functional groups amides I and II
1450–1260	Proteins and lipids with functional groups CH2 and CH3 and phosphate compounds with
	the functional group *P* = O
1260–1180	Polysaccharides C–O–C and C-O-P functional groups
1180–875	-C-O stretching and CH2-bending (peak at 1150 cm-1);
	-C-O stretching (peak at 1070 cm-1); C-H bending (peak at 810 cm-1)
740–580	C = C (alkene) bending (peak at 730–600 cm-1)

### Histological study

**Plate 1:**
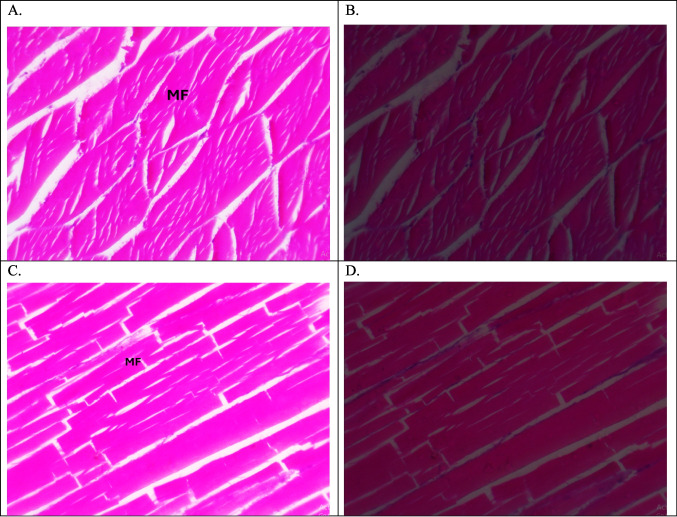
Photomicrographs of muscle of tilapia, *Oreochromis niloticus*, (**A**&**B**) T.S. and (**C**&**D**) L.S. representing negative microplastics samples showing no MPs and displaying the muscle fibers (MF). Under light microscope (LM) (**A** &**C**) and polarizing microscope (PM) (**B**&**D**), (H & E staining, X400)

**Plate 2:**
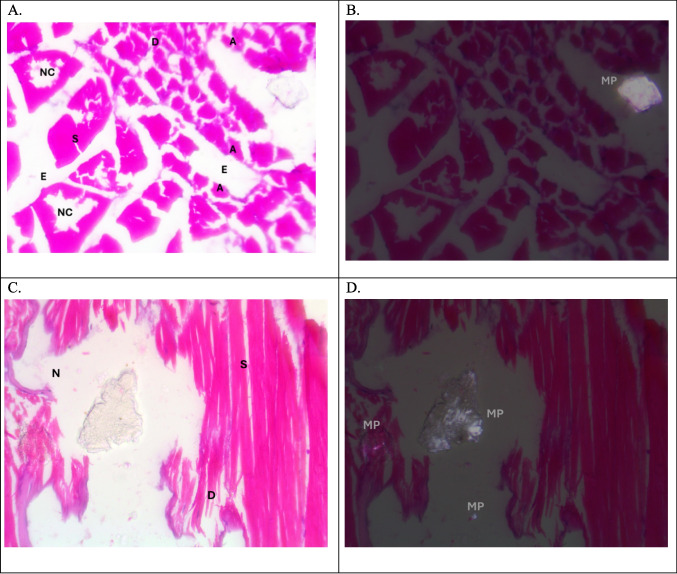
Photomicrographs of muscle of tilapia, *Oreochromis niloticus,* in summer season. (**A**&**B**) T.S. in the Riah El-Towfiqi, and (C&D) L.S. in the Nile. Both represent positive microplastics samples, under light microscope (LM) (**A** &**C**) and polarizing microscope (PM) (**B**&**D**) showing MPs. In (A&C) dissociation (D), segmentation (S), atrophy (A), necrotic core (NC), necrosis (N) and edema (E). (H & E staining, X400)

**Plate 3:**
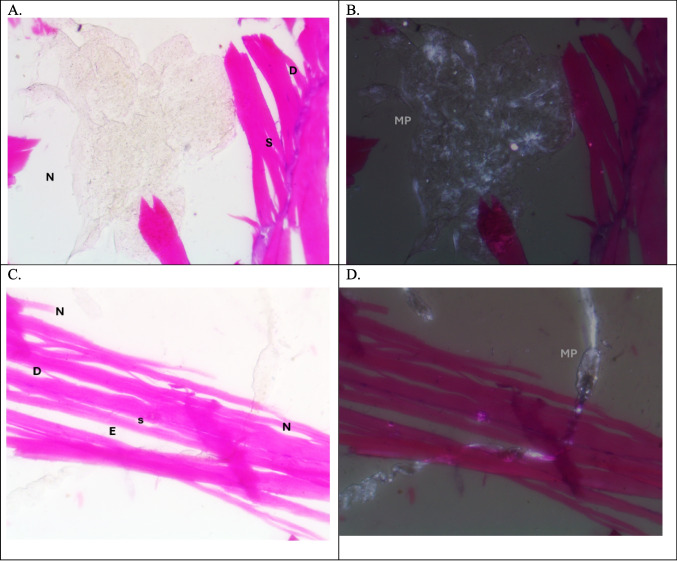
Photomicrographs of muscle of tilapia, *Oreochromis niloticus,* in autumn season. (**A**&**B**) L.S. in the Riah El-Towfiqi, and (**C**&**D**) L.S. in the Nile. In (A&C) dissociation (D), segmentation (S), atrophy (A), necrosis (N) edema (E) (H & E staining, X400)

The muscles are composed of regular polygonal muscle fibers. Each one contains myofibrils with peripherally located nuclei and with tiny unstained area with intermuscular connective tissue. Plate 1A, B, C, D and E represents the negative groups of MPs where the muscle fibers seem to be cylindrical in shape without any histopathological changes. While in positive MPs groups and due to the presence of MPs, the muscles showed many seasonal histopathological changes, at the studied sites. However, during summer and autumn, there were dissociations, necrosis” necrotic core” and segmentation of muscles fibers, while in the Riah El-Towfiqi there were edema, and atrophy (Plate 2 and 3: A, B, C & D). In winter, muscles showed the same previously mentioned histopathological alterations.

**Plate 4:**
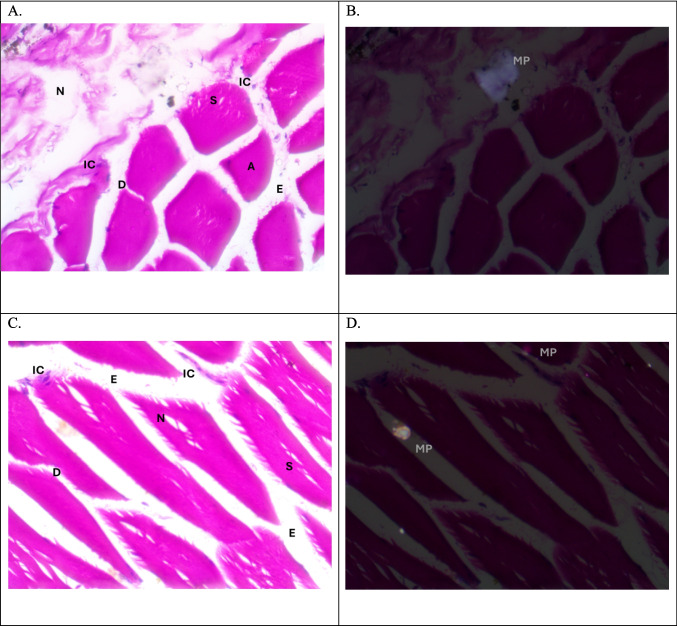
Photomicrographs of muscle of tilapia, *Oreochromis niloticus,* in winter season. (**A**&**B**) T.S. in the Riah El-Towfiqi, and (**C**&**D**) L.S. in the Nile. In (A&C) dissociation (D), segmentation (S), atrophy (A), necrosis (N) edema (E) and inflammatory cells (IC). (H & E staining, X400)

**Plate 5:**
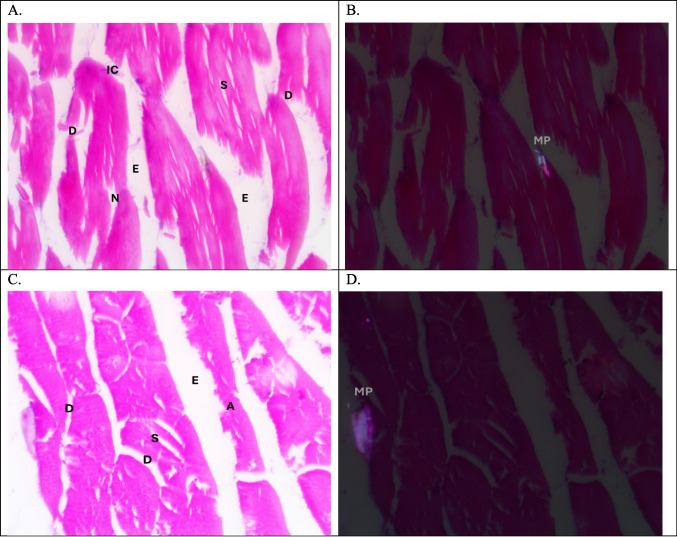
Photomicrographs of muscle of tilapia, *Oreochromis niloticus,* in spring season. (**A**&**B**) L.S. in the Riah El-Towfiqi, and (**C**&**D**) L.S. in the Nile. In (A&C) dissociation (D), segmentation (S), atrophy (A), necrosis (N) edema (E) and inflammatory cells (IC). (H & E staining, X400)

In addition, in the Riah El-Towfiqi the muscles showed atrophy, (Plate 4: A, B, C&D). In spring, there was dissociation, edema and segmentation. Also, there were inflammatory cells and necrosis, while, in the Nile, there was an atrophy of the muscle fiber (Plate 5: A, B, C&D).

### Gene Expression

**Fig. 6:**
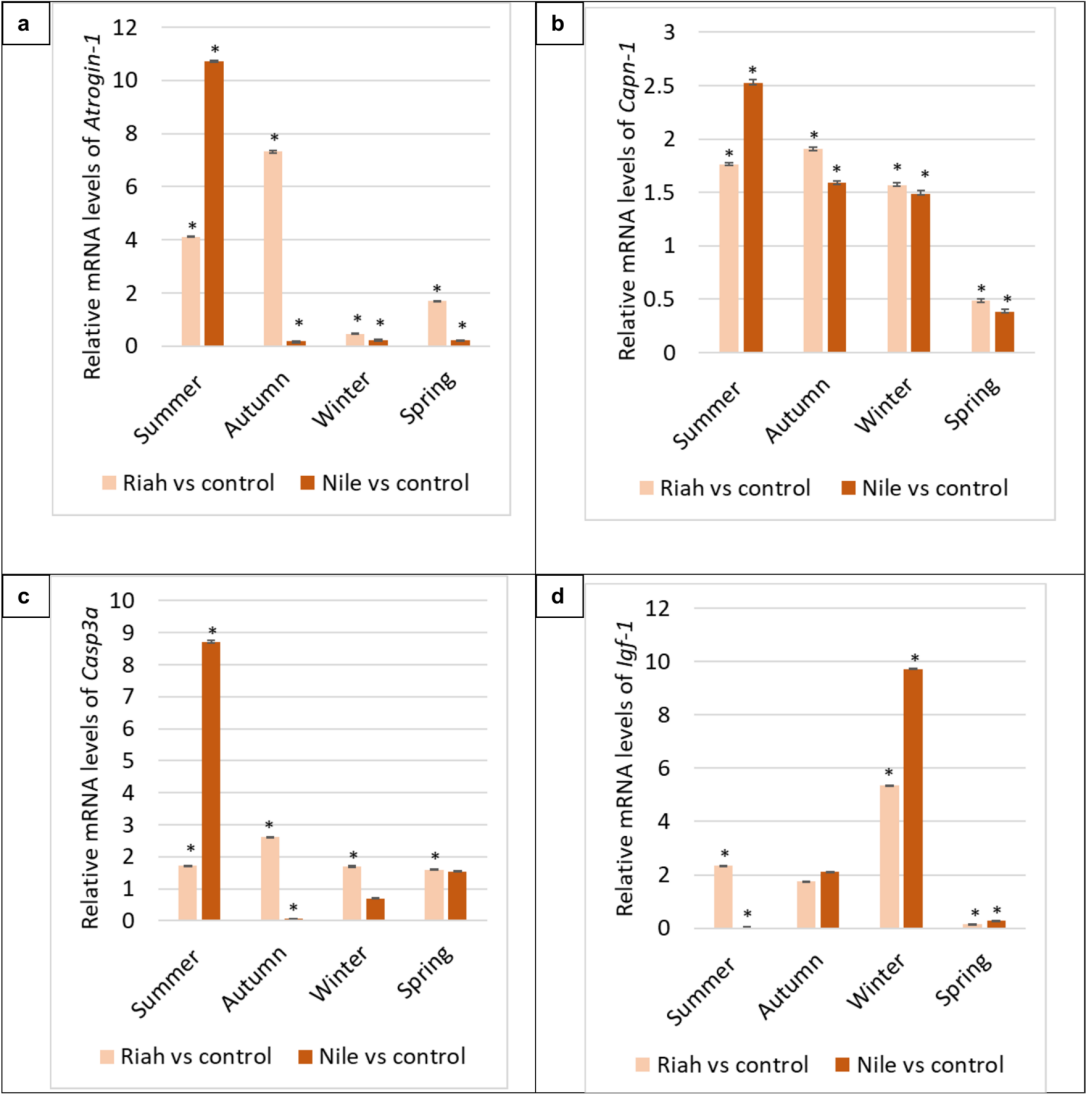

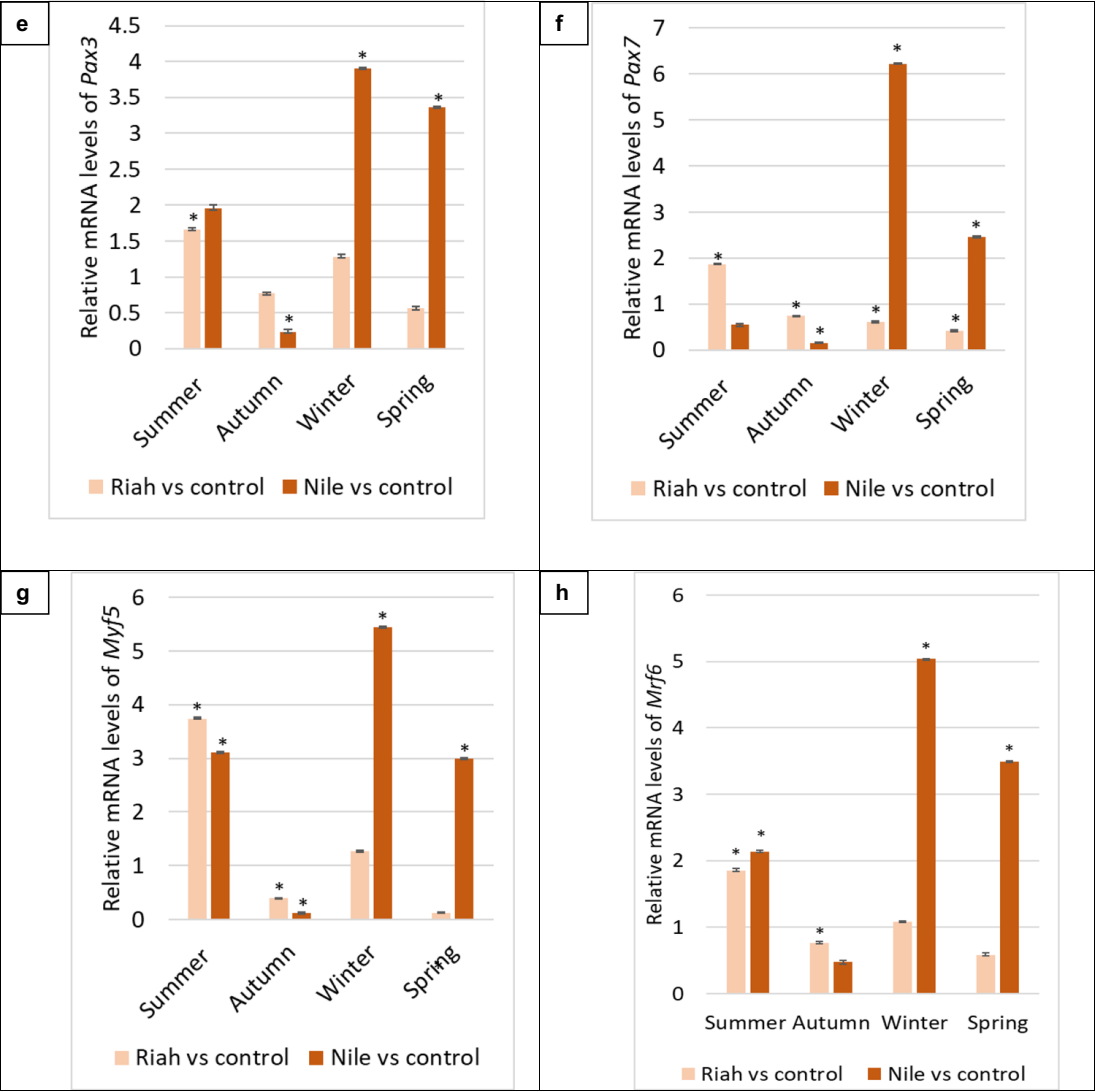
Differential gene expression of a. *Atrogin-1, b. Capn-1, c. Casp3a*, d. *Igf-1*, e. *Pax3,* f. *Pax7,* g. *Myf5* and h. *Mrf6* genes in muscle tissue of fish collected from the Damitte branch and Riah El-Towfiqi during the four seasons. The expression of genes was calculated using fold change between negative control and fish with MPs samples from each place ± standard deviation where (*n* = 16). * is the significance between the affected groups with MPs compared to the negative control at *p* ≤ 0.05

The results showed no significant variation (*p* > 0.05) in *β-actin* Ct values between groups, suggesting that its expression remained stable in our specific experimental conditions. The analyzed genes were quantified using qPCR to find out the variations in their expression levels in fish with MPs relative to the negative control (fish without MPs). During the summer season, the *Atrogin-1*, *Capn-1*, and *Casp3a* genes in Riah El-Towfiqi exhibited significant expression in the muscle tissue of fish affected by MPs, with fold changes of 4.10, 1.76, and 1.71, respectively, compared to their negative control groups (*p* ≤ 0.05). In contrast, these genes were significantly expressed in the muscle of fish with MPs from the Nile branch, showing fold changes of 10.70, 2.53, and 8.71, respectively, compared to their negative control groups (*p* ≤ 0.05), as illustrated in Fig. 6.

During the autumn season, in Riah El-Towfiqi, the expression levels of *Atrogin-1*, *Capn-1*, and *Casp3a* genes, as illustrated in Fig. 6, were considerably upregulated in fish muscle by factors of 7.32, 1.91, and 2.61, respectively, in comparison to the negative control group (*p* ≤ 0.05). The expression of *Atrogin-1* and *Casp3a* genes were markedly downregulated in the muscle of fish from the Nile branch groups relative to the negative control group, although calpain-1 expression exhibited a significant increase of 1.59 times (*p* ≤ 0.05). During the winter season, the expression of the *Atrogin-1* gene was markedly downregulated, whereas the *Capn-1* and *Casp3a* genes in fish from Riah El-Towfiqi exhibited significant increases of 1.57 and 1.70 folds, respectively (*p* ≤ 0.05), compared to the negative control group. The expression of *Atrogin-1* and *Casp3a* genes, in the Nile branch fish, was considerably diminished in the fish from the MP + ve groups compared to the negative control group, although *Capn-1* gene expression rose significantly by 1.49-fold (*p* ≤ 0.05).

During the spring season, the expression levels of the *Atrogin-1* and *Casp3a* genes were significantly elevated by factors of 1.71 and 1.59, respectively, whereas the expression of the *Capn-1* gene was significantly diminished (*p* ≤ 0.05) in the Riah El-Towfiqi of fish subjected to microplastic group compared to the negative control. On the other side, in the Nile branch, the expression of *Atrogin-1* and *Capn-1* genes in tilapia muscles was considerably reduced in the microplastic group compared to the negative control groups, although *Casp3a* expression increased by 1.53 times (*p* ≤ 0.05), as illustrated in Fig. 6.

In the Riah El-Towfiqi, during the summer season, the expression levels of *Igf-1, Pax3, Pax7, Myf5,* and *Mrf6* exhibited significant increases in fish exposed to microplastic groups compared to the negative control group, with fold changes of 2.33, 1.66, 1.86, 3.74, and 1.85, respectively (*p* ≤ 0.05). In the Nile branch, *Pax3, Myf5*, and *Mrf6* were significantly elevated by 1.95, 3.10, and 2.14 folds, respectively, while *Igf-1* and *Pax7* were significantly reduced in fish from microplastic group compared to the negative control group (*p* ≤ 0.05), as illustrated in Fig. 6.

During the autumn season, the expression levels of the *Pax3, Pax7, Myf5*, and *Mrf6* genes in the Riah El-Towfiqi were downregulated in fish exposed to MPs compared to the control group. In the Nile branch, the same genes were markedly downregulated in fish exposed to MPs compared to the negative control group. In contrast, in Riah El-Towfiqi, the expression of the *Igf-1* gene exhibited a significant increase of 1.75 fold in the fish subjected to microplastic group compared to the negative control (*p* ≤ 0.05), while *Igf-1* expression increased significantly by 2.10 times (*p* ≤ 0.05), in fish inhibiting the Nile branch.

During the winter season, the expression levels of *Igf-1, Pax3, Myf5*, and *Mrf6* genes in the Riah El-Towfiqi were up-regulated by 5.35, 1.28, 1.26, and 1.09 folds, respectively. Conversely, *Pax7* exhibited a significant downregulation in expression in the fish exposed to MPs compared to the negative control group (*p* ≤ 0.05). In the Nile branch, the expression levels of *Igf-1, Pax3, Pax7, Myf5*, and *Mrf6* genes were significantly elevated in the fish with MPs compared to the negative control group, with increases of 9.72, 3.81, 6.22, 5.44, and 5.04 folds, respectively (*p* ≤ 0.05), Fig. 6.

**Fig. 7:**
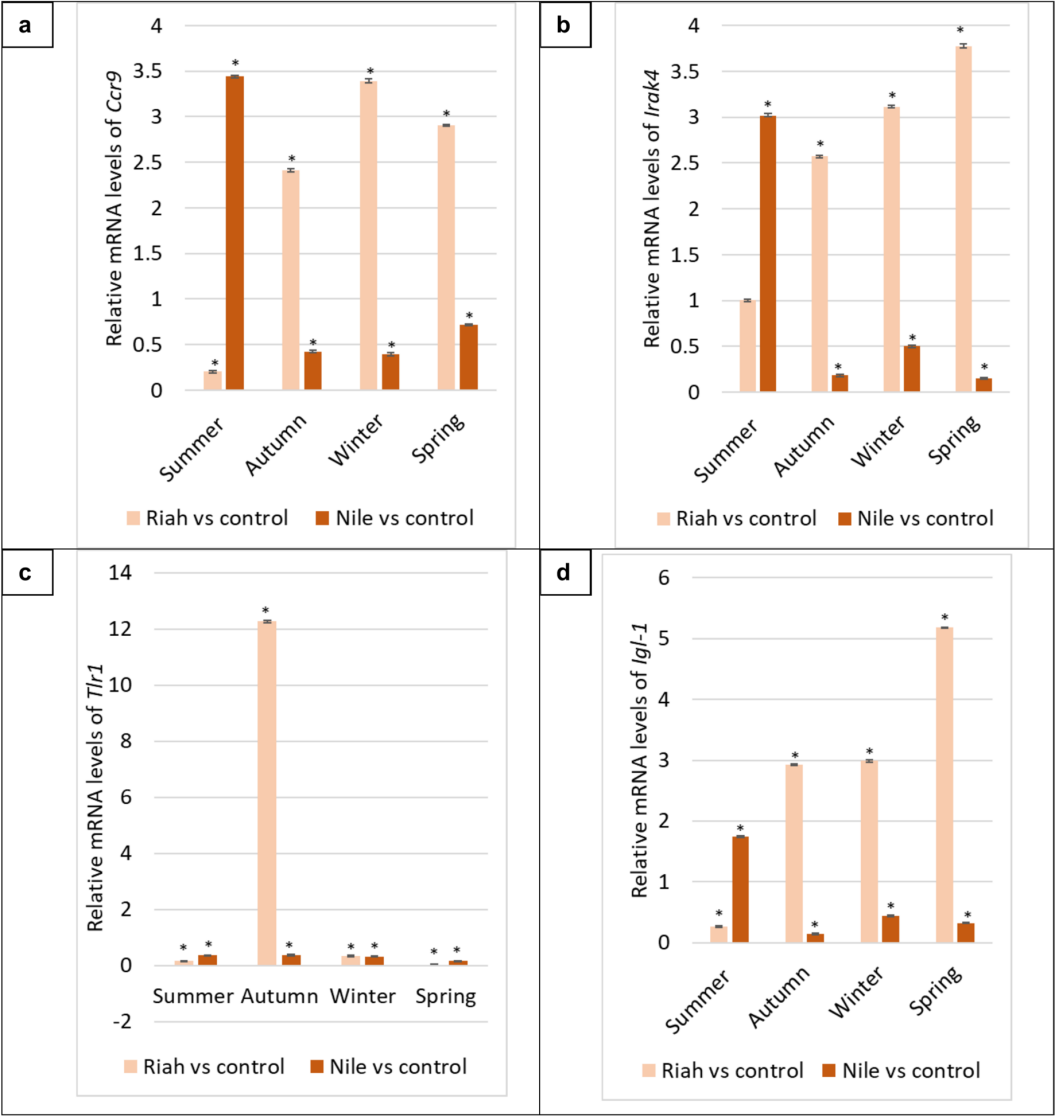
Differential gene expression of a. *Ccr9*, b. *Irka4*, c*. Tlr1* and d. *Igl-1* genes in spleen tissue of fish collected from the Nile branch and Riah El-Towfiqi during the four seasons. The expression of genes was calculated using fold change between negative control and fish with MPs samples from each place ± standard deviation where (*n* = 16). * is the significance between the affected groups with MPs compared to the negative control at *p* ≤ 0.05

During the spring season, the expression levels of *Igf-1, Pax3, Pax7, Myf5*, and *Mrf6* genes in fish from the Riah El-Towfiqi were downregulated in fish exposed to MPs compared to the negative control (*p* ≤ 0.05). Conversely, in the Nile branch, the expression levels of *Pax3, Pax7, Myf5*, and *Mrf6* genes significantly increased in fish with MPs compared to the negative control groups by factors of 3.36, 2.46, 2.99, and 3.49, respectively, while *Igf-1* expression significantly decreased (*p* ≤ 0.05). For immune related genes, during the summer season in the Riah El-Towfiqi, the expression levels of *Ccr9, Tlr1,* and *Igl-1* genes were markedly down-regulated, but *Irka4* levels exhibited an increase of 1.00-fold in the fish exposed to MPs compared to the negative control group (*p* ≤ 0.05). In the Nile branch, the expression levels of *Ccr9, Irka4*, and *Igl-1* were elevated by factors of 3.44, 3.02, and 1.75, respectively, whereas *Tlr1* exhibited significant decreases in fish subjected to the microplastic groups compared to the negative control group (*p* ≤ 0.05), as illustrated in Fig. 7.

During the autumn season in the Riah El-Towfiqi, the expression levels of the *Ccr9, Irak4, Tlr1,* and *Igl-1* genes exhibited significant increases in fish exposed to MPs compared to the negative control group, with fold changes of 2.41, 2.57, 12.27, and 2.93, respectively (*p* ≤ 0.05). Conversely, in the Nile branch, the same genes were significantly downregulated in fish exposed to MPs compared to the negative control group (*p* ≤ 0.05). During the winter season in the Riah El-Towfiqi, the expression levels of the *Ccr9* and *Irak4* genes were significantly up-regulated by 3.39 and 3.11 folds, respectively. Conversely, the *Tlr1* and *Igl-1* genes exhibited significant downregulation in the fish exposed to MPs compared to the negative control group (*p* ≤ 0.05). In the Nile branch, the expression levels of *Ccr9, Irak4, Tlr1*, and *Igl-1* genes were significantly reduced in the fish subjected to MPs compared to the negative control group (*p* ≤ 0.05).

During the spring season, the expression levels of the *Ccr9, Irak4,* and *Igl-1* genes in the Riah El-Towfiqi were upregulated by factors of 2.90, 3.78, and 5.18, respectively, while *Tlr1* levels were downregulated in fish exposed to MPs compared to the negative control (*p* ≤ 0.05). In the Nile branch, the expression levels of *Ccr9, Irak4, Tlr1,* and *Igl-1* genes were significantly reduced in fish exposed to MPs compared to the negative control group (*p* ≤ 0.05), as illustrated in Fig. 7.

## Discussion

This study examined the effects of microplastic (MP) exposure on the muscle and immune systems of Nile tilapia (*Oreochromis niloticus*) collected seasonally from two key fishing regions in Benha City, Al-Qalyubiya, Egypt: the Nile River (Damietta branch) and the Riah El-Towfiqi canal. Although water temperature was not directly measured, seasonal sampling allowed us to consider potential environmental influences, including seasonal variation, on MP accumulation and physiological responses. Previous studies revealed the correlation between the seasonal variations and accumulation of pollutants and their impact on gene expression such as Frapiccini et al., (2021). These findings are consistent with previous studies showing that microplastic exposure can affect growth and immune gene expression (Romano et al. 2020; Tang 2025).

In this study, the histological and histopathological examinations of the muscles of Nile tilapia, *Oreochromis niloticus,* showed various signs of structural deformation that differ seasonally due to presence of MPs and its size. The most noticed histopathological signs of muscles were dissociation, segmentation, atrophy, necrosis, edema and infiltration of inflammatory cells. Hamed et al., (2021) observed nearly the same histopathological changes in the muscles of early juveniles of Nile tilapia, *Oreochromis niloticus*, after exposure to different sizes of MPs. In addition, Yang et al., (2020) also noticed that MPs caused damaging in muscle structure in the larvae of goldfish*, Carassius auratus*. On contrary, Walpitagama et al., (2019) reported that there were no changes in the muscle of zebrafish embryos, *Danio rerio*, after treatment with 3D-printed plastics; instead, it appeared with myoseptal segment integrity and its normal organization.

Gene expressions associated with muscle atrophy demonstrated a direct impact of MPs on muscular decrease. The expression of the *Atrogin-1* gene significantly increased throughout the summer season in tilapia fish taken from the Nile branch compared to previous seasons. The expression of the *Atrogin-1* gene in tilapia from the Riah El-Towfiqi exhibited a significant increase during summer, autumn, and spring, whereas it was markedly downregulated in winter. The expression of the calpain-1 (*Capn-1*) gene increased in summer, autumn, and winter, but it drastically decreased in spring within the Nile branch. At the Riah El-Towfiqi, *Capn-1* gene expression in fish muscle was markedly elevated in autumn, summer, and winter, whereas it was significantly downregulated in spring. Furthermore, the expression of the *Casp3a *gene in fish living in the Nile branch exhibited a significant rise during summer relative to other seasons.

At the Riah El-Towfiqi, *Casp3a *exhibited significant increases throughout all seasons. These data aligned with those reported by Daniel et al. (2020), who indicated that the contamination of MPs in *Fenneropenaeus indicus*, was significantly higher in July–August (monsoon season) relative to other months. This phenomenon may result from weathering effects that hinder the decomposition of MPs, which are predominantly located along coastlines, particularly in densely populated regions, subjected to elements such as sunlight, wind, rocks, and waves, all contributing to the fragmentation of plastic into MPs. Furthermore, it has been ascertained that temperature fluctuations may influence the distribution of white muscle, as Brahmane et al. (2017) indicated that in tilapia larvae, *MyoD* gene expression exhibited a significant rise at 34 °C relative to 25 °C and 30 °C, whereas myostatin gene expression remained constant across all these three temperature conditions. Furthermore, Hassan et al. (2017) investigated the impact of varying temperatures during the summer (36˚C) and winter (14˚C) seasons on the liver and gills of Nile tilapia (*Oreochromis niloticus*) by assessing the gene expression of heat shock proteins (*Hsp70, Hsp27*), oxidative stress genes (*Mt* and *Gst*), and several immune-related genes (*Il-1, Il-8*, and *Tlr7*). *Hsp27*, and *Gst* expression levels were elevated in summer in both organs and exhibited similar expression levels in winter. *Hsp70* expression elevated in both organs across both seasons, likely due to its crucial role in ensuring fish survival; however, no alterations in the gene expression of *Mt, Il-1, Il-8,* and *Tlr7* are noted in either season.

MPs have been identified as inducing oxidative stress in fish (Subaramaniyam et al. 2023). Moreover, numerous studies have demonstrated that oxidative stress can induce muscular atrophy via various mechanisms: (1) Upregulation of critical proteolytic proteins, including the muscle-specific E3 ligases such as ATROGIN-1; (2) release of cytosolic calcium ions, which activate *Capn-1* and *Casp3a*; (3) oxidative conformational alterations in myofibrillar proteins, rendering them more vulnerable to degradation due to oxidation, resulting in their unfolding into the primary structure. Consequently, the peptide bonds are exposed and can be cleaved, leading to protein breakdown (Ngo et al. 2013; Powers et al. 2016).

Additionally, Shengchen et al. (2021) investigated the impact of two sizes of polystyrene MPs (PS-MPs, 1–10 µm and 50–100 µm) on the growth and regeneration of anterior tibial muscle following injury in mice. Delays in skeletal muscle regeneration were documented due to the existence of PS-MPs, irrespective of their size, which was ascribed to the overproduction of reactive oxygen species (ROS). The overproduction of ROS impedes muscle regeneration and influences the destiny of satellite cells. Furthermore, ROS induces the overexpression of muscle-specific E3 ligases by inhibiting the activation of Akt and mTOR1 pathways. These alterations impede translation, resulting in muscular atrophy (Powers et al. 2016).

In vitro, exposure of C2C12 myotubes to ROS resulted in a considerable upregulation of genes encoding several proteasome system proteins, including muscle-specific E3 ligases such ATROGIN-1 and CAPN-1 (Powers et al. 2016). Furthermore, human cultured skeletal-muscle satellite cells exhibited a notable elevation in *Capn-1 and −2* upon exposure to H_2_O_2_, which was facilitated by an increase in cytosolic calcium ions (Powers et al. 2016). It is also observed that ROS activate the *Casp3a* gene in skeletal muscles when C2C12 myotubes are exposed to hydrogen peroxide (H₂O₂) (Powers et al. 2016). *Casp3a* is integral to muscle degradation, while oxidative stress induces muscle atrophy by activating *Casp3a*, resulting in enhanced protein catabolism (Powers et al. 2016).

The results of the present study demonstrated a substantial increase in *Igf-1* gene expression in tilapia muscle samples obtained from the Damietta branch throughout winter and autumn, relative to their respective negative control fish. Nonetheless, *Igf-1* exhibited a marked downregulation during summer and spring in comparison to the negative control fish. The expression of the *Igf-1* gene in the muscle of tilapia fish taken from the Riah El-Tawfiqi was highly elevated in winter, summer, and autumn, while it was dramatically downregulated in spring compared to their respective negative control fish.

In muscle tissue, mechanical stress and contraction cause the production of IGF-1, which subsequently binds to its receptor (IGF-1R) on the muscle cell (Barclay et al. 2019). This binding obstructs phosphorylation, resulting in the recruitment of insulin receptor substrate 1 (IRS1) and the activation of the phosphoinositide-3-kinase (PI3K) pathway (Egerman and Glass 2014; Powers et al. 2016). The PI3K activates AKT, and the activation of the IGF1/AKT pathway promotes protein synthesis by augmenting the translation of particular mRNAs (Powers et al. 2016). Multiple studies have indicated that the deactivation of the IGF1 receptor impedes muscle growth (Schiaffino et al. 2013). Moreover, oxidative stress has been documented to obstruct the IGF1/AKT pathway, potentially hindering muscle development (Powers et al. 2016).

The *Pax3* gene in tilapia muscle obtained from the Nile branch exhibited a notable rise during winter, spring, and autumn, whereas it was diminished in summer. Conversely, in muscle fish obtained from the Riah El-Tawfiqi branch, the *Pax3* gene exhibited a notable elevation during both summer and winter.

The expression of the *Pax7* gene in the muscles of fish from the Nile branch exhibited a considerable rise in winter and spring, but it was downregulated in autumn and summer. The expression of the *Pax7* gene was markedly elevated in summer in the tilapia muscle obtained from the Riah El-Tawfiqi compared to the negative control fish. These findings align with prior studies that emphasized the significance of *Pax7* and its paralog *Pax3* in myogenesis. The augmentation of fish muscle mass results from the involvement of myogenic stem cells, termed myosatellite cells, situated external to the basal lamina of muscle fibers (Kacperczyk et al. 2009). Upon recruitment and proliferation, myosatellite cells fuse with adjacent muscle fibers (Koumans and Akster 1995), leading to muscular hypertrophy and hyperplasia (Kacperczyk et al. 2009). Relaix et al. (2006) established that the *Pax3* gene is expressed in both dormant and active satellite cells across different skeletal muscles. *Pax3* is crucial for the migration of progenitor cells to future skeletal muscle regions and is vital for their survival (Buckingham and Relaix 2015). This is corroborated in *Pax3* mutant mice, whose muscle precursors could not migrate, resulting in the loss of limbs and specific head muscles due to defective dermomyotome development (Buckingham and Relaix 2015).

Furthermore, in *Pax7* mutants, satellite cell populations are markedly diminished in muscles, irrespective of *Pax3* expression. The decrease was ascribed to the demise of satellite cells, impacting the cell cycle (Buckingham and Relaix 2007; Relaix et al. 2006). The expression of *Pax1, Pax3*, and *Pax7* in the somatic mesoderm is crucial for the specification of the sclerotomal and dermomyotomal, as demonstrated by deficiencies in *Pax1* and *Pax3* mutants (Tremblay and Gruss 1994). The *Pax3* and *Pax7* proteins govern late myogenic cells, which differentiate into satellite cells essential for postnatal muscle development and regeneration (Hammond et al. 2007; Relaix et al. 2006).

The *Myf5* gene exhibited considerable overexpression in the muscle of tilapia from the Nile branch throughout winter, summer, and spring compared to the negative control fish for each respective season; however, a marked decrease was observed in autumn relative to its negative control fish. In the Riah El-Tawfiqi, *Myf5* exhibited a substantial rise in summer and a negligible increase in winter relative to the negative control. Nonetheless, it was markedly reduced in autumn and spring compared to the negative control groups.

This study emphasizes the significance of the *Myf5* gene in muscle growth. Consistent with other research indicating that the lack of the *Myf5* gene leads to delayed myotome formation, with recovery contingent upon the expression of *Myod* (Borycki et al. 1999; Tajbakhsh and Rocancourt 1997), it is suggested that *Myf5* is a crucial gene in early myogenic determination. *Myf5* has consistently been recognized as an onset gene activated in both epaxial and hypaxial muscles of the mouse embryos at embryonic stage day 8.5 (E8.5) (Borycki et al. 1999).

The expression of the *Mrf6* gene in fish muscle samples from the Nile branch exhibited a significant increase during winter, spring, and summer compared to the negative control, whereas during autumn it was non-significant decrease. At the Riah El-Tawfiqi, *Myrf6* expression significantly increased in summer, with a non-significant fluctuation in winter, autumn and spring compared to their respective negative control fish. The findings aligned with Kassar-Duchossoy et al. (2004), which indicated that *Mrf6*, or *Mrf4* as referenced by Zhou et al. (2018), is the inaugural gene expressed throughout muscle development in *Myf5/Myod* double-null mice. Furthermore, Aránega et al. (2021) noted that *Mrf6* facilitates the reorganization of myofilaments and the translocation of central nuclei, hence enhancing the maturation of myotubes. In same pattern, Zanou and Gailly (2013) asserted that the expression of the MRF family, encompassing the myogenic lineage transcription factors (*Myf5, Myod*, myogenin, *Mrf6*, and *Mrf4*), is regulated by *Pax* genes, specifically *Pax3* and *Pax7*.

It has been documented that MPs elicit immunological responses via oxidative stress, leading to the generation of ROS (Schieber and Chandel 2014; Smith et al. 2018; Subaramaniyam et al. 2023). Ahmadifar et al. (2021) assessed the gene expression of interleukin 8 (*Il8*), interferon-gamma (*Ifn-Γ*), interleukin 1 beta (*Il-1β*), and tumor necrosis factor alpha (*Tnf-Α*) in Nile tilapia (*Oreochromis niloticus*) following exposure to polystyrene microparticles, noting substantial increases in the MP groups relative to the control group.

For the *Ccr9* gene, findings from fish inhabit the Nile branch indicated an important increase in summer and a significant reduction in other seasons relative to their respective negative controls. Conversely, in the Riah El-Tawfiqi, the *Ccr9* gene exhibited a significant decrease in summer and a significant increase in other seasons relative to their control groups. These results align with prior research that assessed *Ccr9* in several inflammatory circumstances, indicating elevated expression of the *Ccr9* gene and an increase in T cells during intestinal inflammation (Galindo-Villegas et al. 2013). Likewise, Eksteen et al. (2004) demonstrated an augmented recruitment of *Ccr9*-expressing T cells in individuals with inflamed liver.

In the Nile branch, *Irak4* gene expression significantly increased in summer, whereas it drastically decreased in other seasons (*p* ≤ 0.05). In contrast, in the Riah El-Towfiqi branch, *Irak4* exhibited a non-significant increase in summer and a significant upregulation in other seasons compared to the negative control group. These results align with prior research indicating that MPs function as a barrier to bacteria (Liu et al. 2019; Reichert et al. 2019). Moreover, prior research indicated that *Irak4* (*Onirak4*) and *Irak1* (*Onirak1*) serve as essential antibacterial agents in safeguarding Nile tilapia from *Streptococcus agalactiae* infection (Han et al. 2020). Furthermore, the inhibition of *Irak4* in both mice and humans resulted in significant pyogenic bacterial infections (Cushing et al. 2017).

*Igl-1* gene expression was quantified in fish at the Nile and Riah El-Towfiqi. In the Nile branch, it was significantly higher during summer, whereas it was significantly downregulated in other seasons relative to the negative control. In contrast, in the Riah El-Tawfiqi, *Igl-1* was considerably downregulated in summer and significantly upregulated in other seasons relative to their negative controls. The study on rainbow trout fish, *Oncorhynchus mykiss*, indicated that exposure to small polystyrene MPs (PS-MPs) (0.83–3.1 μm) resulted in a significant reduction of non-phagocytic developing B cells, which correlated with decreased *Rag1* gene expression and alterations in the membrane configuration of immunoglobulin heavy chains, (mu and tau), (Zwollo et al. 2021). Furthermore, they discovered that larger PS-MPs (> 6.8 μm) exhibited reduced interaction with developing B cell viability compared to smaller PS, indicating that larger PS may influence B cells through alternative mechanisms that disrupt cell–cell interactions.

The expression of the *Tlr1* gene in tilapia spleen samples from the Nile branch showed a significant decrease throughout all seasons compared to negative control groups. In contrast, the Riah El-Tawfiqi observed significant gene over expression during the autumn season, whilst other seasons demonstrated significant decreases compared to the negative control groups. Palti (2011) contends that the ligand for TLR1 in fish is yet to be identified. Moreover, Quiniou et al. (2013) noted that in channel catfish, *Ictalurus punctatus*, TLR1 is devoid of the LRRNT domain, essential for the identification of pathogen-associated molecular patterns (PAMP). Thus, it is proposed that TLR1 interacts with TLR2, leading to dimerization similar to that seen in mammals (Quiniou et al. 2013; Zhang et al. 2014). The results of the present study align with those of Cao et al. (2023), who reported a significant increase in *Tlr2* expression in murine lung tissue exposed to small (1–5 μm) and large (10–20 μm) PS-MPs. Also, *Tlr1* expression significantly increased in treated murine lung tissue with small MPs (1–5 μm). But, in A549 cells, the *Tlr1* showed higher expression levels in case of larger MPs (10–20 μm) than smaller ones (1–5 μm) (Cao et al. 2023).

## Conclusion

The data obtained in this study reveald distinct effects of microplastics (MPs) on Nile tilapia (*Oreochromis niloticus*), with variations observed between the Nile River (Damietta branch) and Riah El-Towfiqi. These site-specific differences in the expression of muscle-related and immune-related genes may be attributed to seasonal and habitat-related environmental variabilities. The findings highlighted the physiological stress responses in fish due to MPs exposure, suggesting potential ecological risks to aquatic organisms in microplastic-contaminated environments. This study contributes to a growing body of evidence on MPs-induced molecular toxicity in River Nile tilapia and supports the need for further investigation into MPs pollution and its regulatory oversight.

## Data Availability

No datasets were generated or analysed during the current study.
